# Dual Epidemics of Club Drug Use and Sexually Transmitted Infections among Chinese Female Sex Workers: New Challenges to STI Prevention

**DOI:** 10.1155/2017/2093421

**Published:** 2017-05-30

**Authors:** Jing Li, Xiang-dong Gong, Xiaoli Yue, Ning Jiang

**Affiliations:** Institute of Dermatology, Chinese Academy of Medical Sciences and Peking Union Medical College, Nanjing 210042, China

## Abstract

**Objectives:**

To evaluate club drug use and its potential association with STI among female sex workers (FSWs) in China.

**Methods:**

From November 2008 to January 2009, participants were recruited at sex work venues in five cities for a questionnaire survey. Free testing for syphilis,* Chlamydia trachomatis* (CT), and* Neisseria gonorrhoeae* (NG) was provided. Logistic regression models were used to assess factors associated with club drug use and its association with STI.

**Results:**

A total of 1604 eligible FSWs were included. The overall prevalence of any STI infection and club drug use in the past 12 months was 22.6% and 7.4%, respectively. STI prevalence was significantly higher among club drug users (33.1%) than among nonusers (21.7%, *P* < 0.05). Multivariable logistic regression found that club drug use was associated with younger age (AOR 2.4, 95% CI 1.0, 6.0), higher education, having injected drugs (AOR 24.4, 95% CI 6.2, 96.8), and having had STI symptoms (AOR 2.2, 95% CI 1.4, 3.4).

**Conclusions:**

Club drug use and STI were highly prevalent among FSWs in China, especially among young FSWs. Club drug users had more risk behaviors and higher STI rates. A coordinated risk reduction framework is urgently needed to address the dual epidemic of drug use and STI.

## 1. Introduction

Abuse of club drugs which refer to a group of synthesized drugs like methamphetamine and ecstasy has been on the rise around the world [1, 2]. Club drug users can have heightened sex drive, prolonged sexual intercourse, and increased number of sex acts [3, 4]. Studies in western countries showed that club drug use was associated with high-risk sexual behaviors and elevated risk of HIV and other sexually transmitted infections (STIs) among different risk groups [5, 6].

Like western countries, China has also seen a boom of club drug use in the past two decades [7, 8]. Club drugs are rapidly replacing heroin and becoming the most widespread illicit drugs in China [8, 9]. Of the newly identified drug users in 2014, over half had used club drugs [9]. Given the predominant contribution of sexual transmission to HIV/STI epidemic in China [10], the use of club drugs among female sex workers (FSWs) and other risk groups could potentially fuel the epidemic. Recently, several Chinese studies reported the extent of club drug use among men who have sex with men [11, 12]. And one available study conducted among FSWs in a northeastern city in China found an association between club drug use and syphilis infection [13, 14]. Nevertheless, the prevalence and patterns of club drug use differ across populations and regions, and studies assessing club drug use and its association with HIV/STI risk remain scarce in China.

In the current study, we conducted a survey among FSWs in five cities in China to understand the scale of club drug use and assess its association with STI risk. The findings can provide evidence for designing and implementing more targeted sexual risk reduction interventions for FSW.

## 2. Method

### 2.1. Study Setting and Survey Procedure

The survey was conducted alongside routine sentinel surveillance work and the detailed procedure was reported elsewhere [15]. Briefly, between November 2008 and January 2009, a biobehavioral survey was conducted among FSWs at five national STI sentinel surveillance sites to reflect the geographic diversity in China. The five sites included Changchun in Jilin Province (northeast China), Lanzhou in Gansu Province (northwest China), Wuhan in Hubei Province (central China), Hangzhou in Zhejiang Province (east China), and Guangzhou in Guangdong Province (south China).

Health workers at sentinel sites mapped and routinely updated a list of sex work venues including information on venue types and the estimated number of FSWs at each venue. About 40 venues stratified by venue types were randomly selected from the list at each study site. If venue gate-keepers declined to participate, we would go to the next same-type venue on the list as a replacement. A maximum of 20 participants were included at each venue. Eligible participants were women who were 16 years old or above, self-reported to have exchanged sex for money in the past year, and were willing to undergo STI testing. After giving informed consent, participants were interviewed anonymously using a structured questionnaire to gather data on demographics and risk behaviors. All interviews were done in separate rooms or private space to ensure confidentiality. A small token (20 yuan, about 3 US dollars) was given as an incentive to those who agreed to take part in the survey.

The study was reviewed and approved by the Medical Ethics Committee of The Chinese Academy of Medical Sciences Institute of Dermatology and National Center for STD Control in Nanjing.

### 2.2. STI Testing

Free STI tests were provided to all participants. For syphilis testing, blood samples were collected and first tested for treponemal antibody using an enzyme-linked immunosorbent assay (ELISA). Positive ELISA tests were confirmed by nontreponemal toluidine red unheated serum test (TRUST, Rongsheng Biotechnology Company, Shanghai, China). Syphilis tests were performed at study sites according to the national algorithms and an external quality assurance procedure was in place to ensure favorable performance. Endocervical swabs were collected and tested for* Neisseria gonorrhoeae* (NG) and* Chlamydia trachomatis *(CT) by polymerase chain reaction (PCR, AMPLICOR, Roche, USA). The PCR tests were performed at National STD Reference Laboratory in Nanjing. All participants were informed of their syphilis testing results within a week and NG and CT results sometime later. Participants with positive results were referred to designated clinics for counseling, further evaluation, and possible treatment according to the national guidelines.

### 2.3. Measures

Club drug use was the primary outcome of interest. In the survey, FSWs were asked whether they had used methamphetamine, ketamine, and ecstasy/MDMA or to specify other club drugs if any in the past 12 months. Those who answered “yes” to one of the above drugs were coded as club drug users, while those who answered “no” to all drugs were coded as nonusers. For calculating prevalence, syphilis infection was defined as having positive results of both ELISA and TRUST tests, while CT or NG infection was defined as having a positive PCR testing result. STI infection was defined as having syphilis or CT or NG infection.

### 2.4. Data Analysis

All data from questionnaires and laboratory records were double-entered and checked for consistency using EpiData software. Chi-square tests were used to compare differences in club drug use and STI rates among different groups of FSWs. The variables associated with club drug use and STI were assessed first using univariate logistic regression models. Factors significant in univariate analysis (*P* < 0.10) were then assessed in multivariable regression analysis with backward stepwise elimination. All data were analyzed using Statistical Program for Social Sciences (SPSS, version 13.0, Chicago, IL) software.

## 3. Results

Among 1610 FSWs enrolled, 1604 provided cervical swabs and were included in the study. Of all participants, 356 FSWs were enrolled in Guangzhou, 308 in Hangzhou, 243 in Wuhan, 349 in Lanzhou, and 348 in Changchun. Overall, 118 (7.4%) reported club drug use in the past 12 months. The frequency of reported club drug use was as follows: ketamine (77/1604, 4.8%), methamphetamine (72/1604, 4.5%), ecstasy/MDMA (30/1604, 1.9%), and other drugs including marijuana and triazolam (5/1604, 0.3%). The prevalence of any STI infection (syphilis or CT or NG positive) was 22.6% (326/1604). The prevalence of syphilis, NG, and CT infection was 5.4% (86/1604), 5.5% (89/1604), and 13.8% (222/1604), respectively.

Table 1 shows the difference of demographic and behavioral characteristics between club drug users and nonusers. Compared with nonusers, club drug users were more likely to be younger, unmarried, better educated, recruited from Guangzhou, based in night clubs or KaraOK, have injected drugs, and have STI diagnosis in the past 12 months.

Table 2 shows the factors associated with club drug use. In univariate analysis, factors like study sites, types of sex work venue, age, marital status, education level, injection drug use, and history of STI were significantly associated with club drug use. In multivariable analysis, factors retained significant association with more club drug use included being recruited from Guangzhou (adjusted odds ratio (AOR) 3.2, 95% confidence interval (CI) 1.8, 5.8) and Wuhan (AOR 3.5, 95% CI 1.8, 7.0) relative to Hangzhou, being based in night clubs or KaraOK relative to saunas (AOR 2.8, 95% CI 1.5, 5.1), aged under 20 versus above 30 years (AOR 2.4, 95% CI 1.0, 6.0), having middle (AOR 5.1, 95% CI 1.5, 17.4) or high school education (AOR 6.8, 95% CI 1.9, 23.6) versus primary school education, having injected drugs (AOR 24.4, 95% CI 6.2, 96.8), and having had STI symptoms (AOR 2.2, 95% CI 1.4, 3.4) in the past 12 months.

Table 3 shows univariate and multivariate analysis of the factors correlated with STI infection. STI prevalence was significantly higher among FSWs who used club drugs in the past 12 months (33.1%) than those who did not (21.7%, *P* < 0.05). STI prevalence was highest among FSWs in Guangzhou (31.2%) and Hangzhou (26.9%), followed by Wuhan (24.3%), Lanzhou (19.2%), and Changchun (12.1%, *P* < 0.001). Other factors significantly associated with higher STI prevalence in univariate analysis included age, marital status, education level, and types of sex work venue. After controlling for demographic and sex work factors, the association between club drug use and STI was no longer significant in multivariable analysis. In the final model, factors associated with higher STI risk included being recruited from Guangzhou (AOR 2.9, 95% CI 1.8, 4.5), Hangzhou (AOR 2.5, 95% CI 1.6, 3.8), Wuhan (AOR 2.2, 95% CI 1.4, 3.4), and Lanzhou (AOR 2.0, 95% CI 1.3, 3.1) relative to Changchun, being based in hair salons or rental houses (AOR 1.8, 95% CI 1.3, 2.5) relative to saunas, aged under 20 (AOR 1.6, 95% CI 1.1, 2.4) versus above 30 years, and having no more than primary (AOR 1.7, 95% CI 1.1, 2.6) or middle school education (AOR 1.6, 95% CI 1.2, 2.2).

## 4. Discussion

The prevalence of club drug use and its correlation with sexual risk taking are important issues that have not been closely studied among FSWs in China. Understanding the extent and health effects of club drug use might help identify new modifiable factors that affect FSW's vulnerability to HIV/STI. We conducted a study in five cities in different parts of China to explore the prevalence of club drug use and its association with STI among FSWs. The current study confirms that club drug use is common among FSWs in China. In our study, over 7% of FSWs reported club drug use during the past year, which is lower than life-time use among FSWs in Shandong province of China [13, 14]. This is probably due to the difference in study locations and design.

The top three most frequently reported club drugs were ketamine, methamphetamine, and ecstasy, which is similar to national patterns and findings among club drug users [9, 16]. We found significant geographic variations in club drug use. FSW in Guangzhou, Wuhan, and Hangzhou reported more club drug use than FSWs in Changchun and Lanzhou. This is generally consistent with the drug transmission routes in China [8, 16]. Additionally, FSWs in Guangzhou, Wuhan, and Hangzhou where local economy is more developed might be able to afford drug use. It could also be due to the fact that club drug use was more prevalent among clients in these areas [9]. Club drug use was more prevalent among younger and better educated FSWs, which is consistent with national patterns and findings of other studies [9, 13, 14, 17, 18]. Club drug use was found to be associated with injecting drug use and history of STI. Other studies have also found that FSWs who used club drugs tend to have more risk behaviors [13, 14, 17, 18]. Prevention programs will need to account for club drug use in shaping risk patterns and design appropriate intervention measures.

We found a high prevalence of syphilis, NG, and CT infection among FSWs and STI prevalence was significantly higher among FSWs who used club drugs (33.1%) than those who did not use them (21.7%). This is consistent with findings of other studies conducted in China and abroad [13, 14, 17, 18]. Club drug use can elevate the risk of HIV/STI through unprotected sexual intercourse, trauma from prolonged intercourse, and less control of condom use under influence [3, 4]. FSWs who use club drugs may be part of a high-risk sexual network. Clients preferring sex without condoms may be more likely to seek out FSWs who use drugs to offer drugs in exchange for unprotected sex [17]. Since HIV is increasingly spreading through sexual activities in China, club drug use could potentially further fuel the epidemic in China if effective timely interventions are not taken. Current intervention efforts should recognize the emerging epidemic of club drug use and increase the understanding of risk associated with club drug use.

We also found that FSWs under age 20 had significantly higher risk of STI. Young FSW may have less knowledge and experience with STI prevention or may be more susceptible to some STIs due to cervical ectopy following sexual initiation [19–21]. On the other hand, when controlling for demographic and sex work factors in multivariable analysis, the association between club drug use and STI was no longer significant. This is probably owing to the concurrent high prevalence of club drug use and STI among young FSWs. Club drugs meet the psychological characteristics of young people (i.e., curiosity and excitement) and are often perceived as of little or no harm [9]. Additionally, untreated STI can increase the risk of HIV acquisition and transmission beside causing serious adverse health outcomes [22]. Therefore, the dual high prevalence of club drug use and STI among young FSWs underscores the urgency to establish a coordinated and better targeted intervention program to jointly address drug use and safe sex issues.

We also found that the types of sex work venues were associated with club drug use and STI. FSWs based at night clubs and KaraOKs reported much higher club drug use, while FSWs at low-end venues like hair salons and rental houses had higher STI rates. Other studies have also found more club drug use among club-based FSWs and higher STI rates among FSWs at low-end venues [14, 17, 21]. This suggests that sex work venues are important focal points for club drug use and STI transmission. Venue-level interventions appropriate for different types of venues are urgently needed to curtail the intersection of club drug use and STI, especially among young FSWs and those based at low-end venues.

Several limitations of this study should be noted. First, a convenient sample design was employed to recruit FSWs at venues, which means that our sample may not be a good representation of the target population. Secondly, the behavioral data were collected through self-report which might be affected by misreporting bias. Overreporting of condom use by FSWs who used drugs could be the probable reason that no statistically significant association was observed between club drug use and condom use with clients. Thirdly, drug use with different types of sexual partners and sex under the influence of drug use were not explored in the study. More studies are needed to fully understand the context and effect of club drug use to better design intervention programs.

In conclusion, we found high prevalence rates of club drug use and STI among FSWs in China, especially among FSWs under age 20. FSW who used club drugs had more risk behaviors and higher STI rates. Club drug use has the potential to accelerate the sexual transmission of HIV/STI in China. Current prevention programs should recognize the potential of an emerging epidemic of club drug use among FSWs and raise the awareness of risks associated with club drug use. A coordinated risk reduction framework to address the intersection of club drug use and unsafe sex is urgently needed, especially those aimed at young FSWs. More studies are needed to further understand the contexts including sexual partnerships and work environments that may shape drug use patterns among FSW in order to design better targeted prevention messages.

## Figures and Tables

**Table 1:** Characteristics of FSW who used or did not use club drugs in five cities in China.

Characteristics	Drug users (*N* = 118)		Nonusers (*N* = 1486)		*χ* ^2^	*P* value
	Number	%	Number	%		
Study site						
Guangzhou	69	58.5	287	19.3	138.676	<0.001
Hangzhou	18	15.3	290	19.5		
Changchun	1	0.8	347	23.4		
Lanzhou	0	0	349	23.5		
Wuhan	30	25.4	213	14.3		
Venue type						
Hotel	20	16.9	145	9.8	56.901	<0.001
Night clubs/KaraOK	63	53.4	387	26.0		
Saunas	16	13.6	557	37.5		
Hair salons/rental houses	19	16.1	397	26.7		
Age group (years)						
16–20	49	41.5	291	19.6	34.639	<0.001
21–29	59	50.0	892	60.0		
30–48	10	8.5	303	20.4		
Ethnicity						
Han	110	93.2	1404	94.5	0.328	0.567
Minority	8	6.8	82	5.5		
Marital Status						
Never married	101	85.6	884	59.5	31.435	<0.001
Ever married	17	14.4	602	40.5		
Education Level						
≤primary school	3	2.5	167	11.2	8.731	0.013
Middle school	73	61.9	842	56.7		
≥high school	42	35.6	477	32.1		
Injected drugs in the past 12 months						
Yes	7	5.9	8	0.5	34.331	<0.001
No	111	94.1	1478	99.5		
Had STI symptoms in the past 12 months						
Yes	73	61.9	752	50.6	5.547	0.019
No	45	38.1	734	49.4		
Had STI diagnosis in the past 12 months						
Yes	15	12.7	75	5.0	12.126	<0.001
No	103	87.3	1411	95.0		

**Table 2:** Factors associated with club drug use among FSWs in five cities in China (*N* = 1604).

Characteristics	Univariate analysis	Multivariate analysis
	OR (95% CI)	AOR (95% CI)
Study site		
Guangzhou	3.9 (2.3, 6.7)	3.2 (1.8, 5.8)
Hangzhou	1.0	1.0
Changchun	0.1 (0.01, 0.4)	0.1 (0.01, 0.5)
Lanzhou	0	0
Wuhan	2.3 (1.2, 4.2)	3.5 (1.8, 7.0)
Venue type		
Hotel	4.8 (2.4, 9.5)	1.1 (0.5, 2.4)
Night clubs/KaraOK	5.7 (3.2, 10.0)	2.8 (1.5, 5.1)
Saunas	1.0	1.0
Hair salons/rental houses	1.7 (0.8, 3.3)	0.8 (0.4, 1.7)
Age group (years)		
16–20	5.1 (2.5, 10.3)	2.4 (1.0, 6.0)
21–29	2.0 (1.0, 4.0)	1.3 (0.6, 3.0)
30–48	1.0	1.0
Ethnicity		
Han	0.8 (0.4, 1.7)	
Minority	1.0	
Marital status		
Never married	4.05 (2.40, 6.83)	
Ever married	1.0	
Education level		
≤primary school	1.0	1.0
Middle school	4.8 (1.5, 15.5)	5.1 (1.5, 17.4)
≥high school	4.9 (1.5, 16.0)	6.8 (1.9, 23.6)
Injected drugs in the past 12 months		
Yes	11.7 (4.1, 32.7)	24.4 (6.2, 96.8)
No	1.0	1.0
Had STI symptoms in the past 12 months		
Yes	1.6 (1.1, 2.3)	2.2 (1.4, 3.4)
No	1.0	1.0
Had STI diagnosis in the past 12 months		
Yes	2.7 (1.5, 4.9)	
No	1.0	

OR, odds ratio; AOR, adjusted odds ratio; CI, confidence interval; STI, sexually transmitted infection.

**Table 3:** Factors associated with sexually transmitted infection among FSWs in China (*N* = 1604).

Characteristics	STI positive	Univariate analysis	Multivariate analysis
	*N* (%)	OR (95% CI)	AOR (95% CI)
Total	362 (22.6)		
Study site			
Guangzhou	111 (31.2)	3.3 (2.2, 4.9)	2.9 (1.8, 4.5)
Hangzhou	83 (26.9)	2.7 (1.8, 4.0)	2.5 (1.6, 3.8)
Changchun	42 (12.1)	1.0	1.0
Lanzhou	67 (19.2)	1.7 (1.1, 2.6)	2.0 (1.3, 3.1)
Wuhan	59 (24.3)	2.3 (1.5, 3.6)	2.2 (1.4, 3.4)
Venue type			
Hotel	42 (25.5)	1.8 (1.2, 2.7)	1.1 (0.7, 1.8)
Night clubs/KaraOK	111 (24.7)	1.7 (1.2, 2.3)	1.2 (0.9, 1.7)
Saunas	93 (16.2)	1.0	1.0
Hair salons/rental houses	116 (27.9)	2.0 (1.5, 2.7)	1.8 (1.3, 2.5)
Age group (years)			
16–20	106 (31.2)	2.0 (1.4, 2.8)	1.6 (1.1, 2.4)
21–29	197 (20.7)	1.1 (0.8, 1.6)	1.1 (0.8, 1.5)
30–48	59 (18.8)	1.0	1.0
Ethnicity			
Han	343 (22.7)	1.1 (0.7, 1.8)	
Minority	19 (21.1)	1.0	
Marital status			
Never married	247 (25.1)	1.5 (1.1, 1.9)	
Ever married	115 (18.6)	1.0	
Education level			
≤primary school	45 (26.5)	1.8 (1.2, 2.7)	1.7 (1.1, 2.6)
Middle school	229 (25.0)	1.6 (1.2, 2.2)	1.6 (1.2, 2.2)
≥high school	88 (17.0)	1.0	1.0
Used club drugs in the past 12 months			
Yes	39 (33.1)	1.8 (1.2, 2.7)	1.3 (0.8, 2.0)
No	323 (21.7)	1.0	1.0
Consistent condom use with clients last month			
Yes	201 (23.7)	1.2 (0.9, 1.5)	
No	161 (21.3)	1.0	

OR, odds ratio; AOR, adjusted odds ratio; CI, confidence interval; STI, sexually transmitted infection.
